# Innovative mapping of skin sensitivity by monofilaments to record the diagnosis and therapeutic follow-up of leprosy

**DOI:** 10.1590/0037-8682-0386-2021

**Published:** 2022-02-25

**Authors:** Fred Bernardes, Filipe Rocha Lima, Marco Andrey Cipriani Frade

**Affiliations:** 1 Universidade de São Paulo, Faculdade de Medicina de Ribeirão Preto, Departamento de Clínica Médica, Divisão de Dermatologia, Ribeirão Preto, SP, Brasil.; 2 Universidade de São Paulo, Faculdade de Medicina de Ribeirão Preto, Hospital das Clínicas, Centro de Referência Nacional em Dermatologia Sanitária com Ênfase em Hanseníase, Ribeirão Preto, SP, Brasil.

**FIGURE 1:**
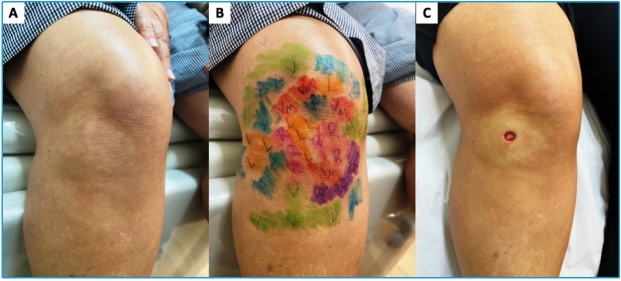
(A) Hypochromic macule on the right knee; (B) altered tactile skin sensitivity mapped by Semmes Weinstein monofilaments ranging from 0.07 gram-force (gf) to 300-gf (normal skin tactile threshold = green monofilament, 0.07-gf); (C) the macule is more evident due to the erythema surrounding the lesion after local anesthesia for skin biopsy, like the incomplete endogenous histamine test. Legend: green (0.07-gf); blue (0.2-gf); violet (2-gf); red (4-gf); orange (10-gf); pink (300-gf).

**FIGURE 2:**
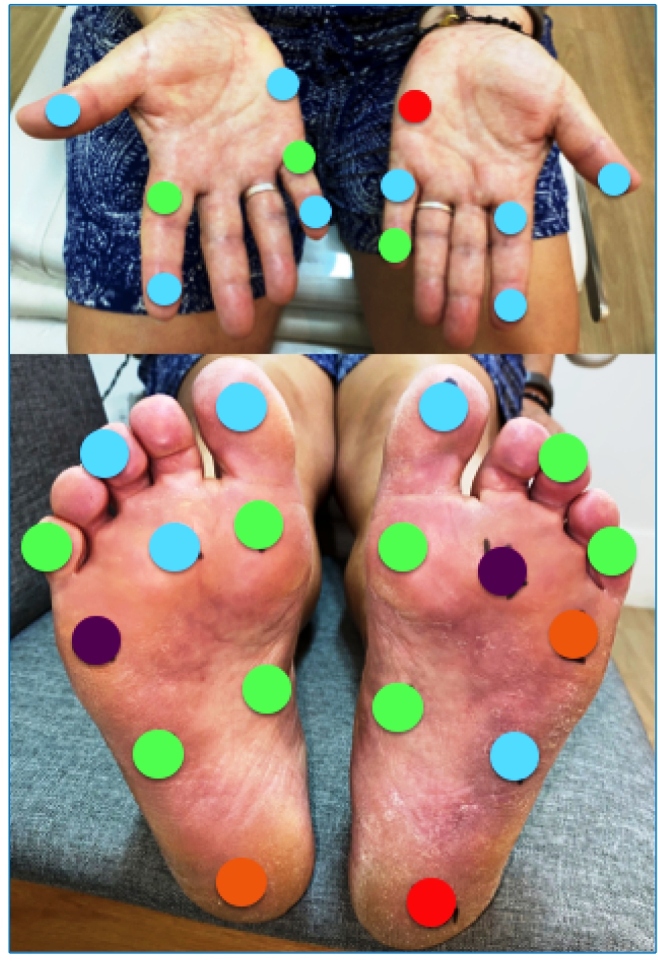
Hands and feet tactile sensitivity at diagnosis. Normal hand tactile threshold = green monofilament, 0.07-gf; normal foot tactile threshold = green and blue monofilaments, 0.02-gf). Legend: green (0.07-gf); blue (0.2-gf); violet (2-gf); red (4-gf); orange (10-gf); pink (300-gf).

**FIGURE 3:**
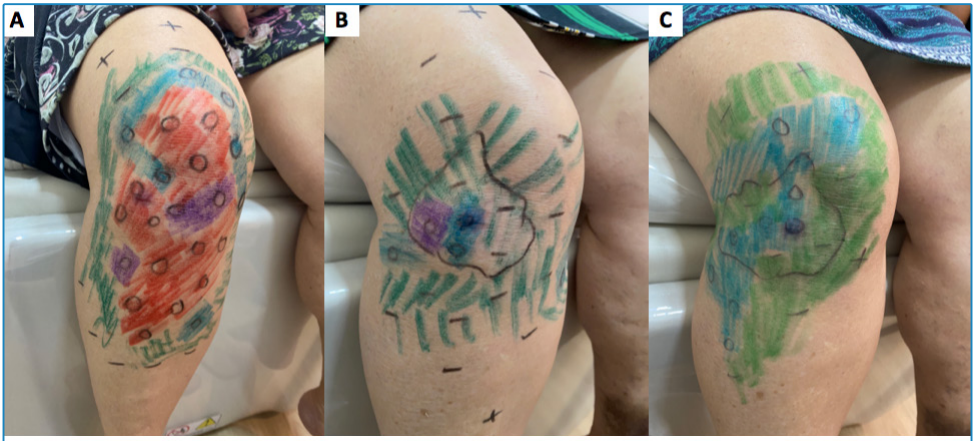
Improvement in skin sensitivity with specific anti-leprosy treatment at the end of the first (a), second (b) and third (c) months. Legend: green (0.07-gf); blue (0.2-gf); violet (2-gf); red (4-gf); orange (10-gf); pink (300-gf).

A 53-year-old previously healthy woman presented with a 5-month history of numbness of the hands and feet, tingling sensation, and nerve pain in the legs. Positive findings on physical examination included hypochromic, anesthetic, and anhidrotic macules, with incomplete endogenous histamine tests on the right knee (Figure 1), electric shock-like pain on common fibular nerves, and altered tactile sensitivity of the hands and feet (Figure 2). She was positive for IgM anti-phenolic glycolipid-I, IgA, and IgM anti-mammalian cell entry 1A protein of *Mycobacterium* antibodies. *Mycobacterium leprae* DNA-specific repetitive element polymerase chain reaction (RLEP-PCR) was positive on skin biopsy and slit skin smear. Ultrasonography of peripheral nerves showed asymmetric and focal multiple mononeuropathies without an intraneural Doppler signal. Borderline leprosy was diagnosed clinically and by laboratory tests, and she was prescribed multibacillary multidrug therapy. Cutaneous lesion mapping (Figure 3) and hands/feet tactile sensitivity tests (Semmes-Weinstein monofilaments) were performed monthly. There was a significant improvement in dermatological signs and neurological symptoms under specific treatment of leprosy with antimicrobials.

Recent studies have demonstrated the importance of appreciating neurological symptoms over dermatological signs in the diagnosis of leprosy^1^. Hypochromic macules with altered sensitivity are a common presentation in the entire spectrum of leprosy^2^ ^,^ ^3^. The exclusive linkage of macular lesions to indeterminate leprosy is an incorrect paradigm in leprosy that leads to inadequate and insufficient treatment. The innovative mapping of cutaneous lesions using monofilaments and the usual hands/feet sensitivity evaluation is an objective assessment that documents the hallmark focality and asymmetry of leprosy at diagnosis and during follow-up.
